# Modeling and Optimization of the Isolation of Blackcurrant and Black Cumin Seeds Oils Using Supercritical Fluid Extraction

**DOI:** 10.3390/molecules27248921

**Published:** 2022-12-15

**Authors:** Barbara Mazurek, Magdalena Wójciak, Dorota Kostrzewa, Małgorzata Kondracka

**Affiliations:** 1 Analytical Department, Łukasiewicz Research Network-New Chemical Syntheses Institute, 24-110 Puławy, Poland; 2 Department of Analytical Chemistry, Medical University of Lublin, 20-093 Lublin, Poland

**Keywords:** SFE, optimization, Box–Behnken design, blackcurrant seeds, black cumin seeds, oil, extraction, RSM

## Abstract

Supercritical fluid extraction is a powerful analytical tool and it is willingly used by researchers for the isolation of various components from different matrices. In our study, the carbon dioxide in the supercritical state was used for the extraction of oils from blackcurrant and black cumin seeds. To determine the optimal conditions for the process (temperature, pressure and time), the method of statistical experiment planning and the Box–Behnken design was applied and the yield of the oils and the content of fatty acids (FAs) were taken into consideration. It has been found that an increase in pressure causes an increase in extraction yield (W), and an increase in temperature, both at constant pressure and time, does not significantly change the yield value. Optimal yield values were obtained for both materials under almost similar extraction parameters: 306 bar/ 43 min/ 50 °C (blackcurrant) and 282 bar/ 40 min/ 50 °C (black cumin). The influence of the above parameters (T, p, t) on the content of FAs in the extracts has a slightly different trend. The use of supercritical carbon dioxide for the extraction of blackcurrant and black cumin seeds allowed for high process yield and high-quality, rich in polyunsaturated fatty acids oils which can be used as a substrate or final product for industry.

## 1. Introduction

Currently, products that are described as “natural” are experiencing a renaissance. Their main sources are primarily plants containing different various bioactive ingredients. They include fiber, fatty acids (especially unsaturated), phytosterols, tocopherols, carotenoids, vitamins and polyphenolic compounds with antioxidant properties. Individually or in groups, these components have a significant impact on the work of the heart and the blood pressure in the cardiovascular system, the digestive system, the skeletal system (including bones and teeth), the body’s lipid metabolism, and its natural resistance (immune system), showing gastro-protective, antithrombotic, anti-inflammatory, antidiabetic, antiviral, antibacterial and antifungal effects [1,2,3,4,5,6,7,8,9,10]. Thus, they are responsible for the health-promoting and/or healing effects of the pharmacological, cosmetic or food products obtained from them.

An important element is therefore the stage of developing effective and selective methods for the isolation of bio-ingredients from natural products. There are several methods to isolate, from plant raw materials, chemical compounds, or mixtures of biologically active compounds showing the desired effects. Different techniques are used depending on what type of compound or group of compounds will be isolated. In addition to conventional methods, such as maceration [11,12], distillation [13], cold pressing [1,14], or Soxhlet and liquid-solid solvent extractions, which require either time or a large amount of organic solvents, more modern, faster and, above all, more ecological methods are currently used. These are, for example, pressurized liquid extraction (PLE), microwave assisted extraction (MAE), ultrasound assisted extraction (UAE), and supercritical fluid extraction (SFE) [15,16,17].

Supercritical fluid extraction is a powerful analytical tool, and it is willingly used by researchers for the isolation of various components from different matrices [18,19]. It allows the obtaining of chemically pure extracts without residues of solvents and heavy metals. The process takes place at relatively low temperatures, in closed systems without access to sunlight and oxygen, which prevents the oxidation, transformation or decomposition of unstable substances. In addition, it is a quick and nearly waste–free extraction, most often using cheap and easily available carbon dioxide as a solvent. 

One of the advantages of the fluid in the supercritical state is its selectivity, which depends primarily on the extraction parameters, as well as on the composition and structure of the plant material. All known processes occurring during SFE extraction were presented by Ramsey et al. (1998) in his book at the end of the 1990s [20], and are currently espoused by Rój E., Pereira C. G., or Ahangari H. [19,21,22]. For bioactive compounds, in the case of SFE, the selectivity is connected with solubility and generally increases with the pressure in the region of its low values, up to its maximum, and then decreases with the further increase in pressure [18,21]. Optimizing the process conditions seems to be the key to the effective extraction of bioactive ingredients from natural plant matrices. 

The purpose of our study was to investigate the supercritical carbon dioxide extraction of oils under different conditions of temperature, pressure and extraction time. Two crop species seeds with different oil content were used as a model. One of them was blackcurrant seeds, a by-product of berry processing, which are unusual for obtaining an oil extract. The second was black cumin seeds, which are a typically oily plant from the buttercup family (Ranunculaceae or crowfoot family). An experimental plan was created for both materials. 

Over the last years, a growing application of the statistical design of experiments is observed as an extremely useful method for the optimization of technological processes [22,23]. To determine the optimal conditions for the process, the method of statistical experiment planning and the Box–Behnken design were used. This method consists of creating a mathematical model based on data obtained from a relatively small number of experiments (several or a dozen experiments). Such a model can be used, for example, to predict the yield of extraction, to determine the optimal values of product parameters, or to determine the parameters of the production process, taking into account the content of selected components in the product expected by the customer and, subsequently, the possibility of transferring it to a larger scale of equipment. Carrying out the process in several experiments on a laboratory scale allows, above all, to reduce financial outlays by reducing the consumption of raw materials, solvents and energy inputs, as well as shortening the time of the process itself, and thus also the time required to wait for the results.

Taking into account the variable extraction parameters such as temperature, pressure, time (T, p, t)–, their mutual dependencies, and the resulting changes in the solubility of the matrix components, it was decided to conduct several supercritical extraction experiments on a laboratory scale using the selected seed material. 

The unique fatty acid composition (high level of polyunsaturated fatty acids in both blackcurrant and black cumin seeds and desirable ω3/ω6 FAs ratio with a high concentration of unique gamma-linolenic acid in black currant), in combination with the high content of lipid-soluble antioxidants, makes these seeds valuable materials. 

The yield (efficiency) of the process and the content of fatty acids (FAs) in the obtained oil extracts were selected for evaluation. 

In the literature, there are some data on the optimization of the black cumin seed extraction process using scCO_2_, and only a small amount of data on the extraction of blackcurrant seeds. In addition, only a few of them indicate the effect of the applied changes in the parameters both on the efficiency of the process and on the content of fatty acids. Moreover, and as far as we know, there are no reports regarding the optimization of FAs content in extracts obtained from blackcurrant and black cumin seeds with scCO_2_ using response surface methodology (RSM). 

It also seems interesting to compare, in this form, the results obtained for the seeds of a typical oily plant with the results for the berry seeds with a different biochemical composition and different climatic requirements. The obtained results and the mathematical description of the parameters correlation presented in the work provide valuable information for other researchers and entities interested in using selected seeds for the purposes of the food, cosmetics, medicine and pharmacy industries.

The obtained results can also be used to transfer the experiment to a larger extraction scale. However, here one should remember the interactions and complicated processes between liquid, gas and supercritical phases, as well the limitations of the laboratory scale equipment, where there is no possibility to isolate water from the obtained extract. 

The use of statistical/mathematical methods allows for a lower total cost of the optimization procedure by the lower consumption of plant material and selection of the optimal working time. Moreover, in relation to the cited literature, selected other values of the conducted experiments parameters cover a fairly wide range of changes, especially in the area of pressure. However, these are not the values of the high pressure area, for which appropriate and often expensive equipment is necessary. Therefore, they can be performed on the equipment available in the laboratory. The proposed time range is also reasonable and allows the research to be carried out during a routine working day.

## 2. Results

### 2.1. Blackcurrant Seeds Extraction

The complete matrix of the experimental design and the results of extraction of blackcurrant seeds with supercritical carbon dioxide obtained during the Box–Behnken design (BBD) implementation are shown in Table 1. The analyzed response variables were: W_CP_—extraction yield (%) and C_FA_—fatty acid content (% *w/w*).

The regression coefficients of equations in the form of second-order polynomials for the yield of extraction of blackcurrant seeds with supercritical carbon dioxide (W_CP_) and for the total content of fatty acids in the extracts (C_FA_) together with basic statistical data describing the correctness of fitting general square models to experimental data are presented in Table 2. 

The *p*-value in the case of a mathematical model in the form of a second–order polynomial for the extraction yield of blackcurrant seeds is 0.0032, and for the content of fatty acids in the extract it is 0.0021. This means that the obtained models are statistically significant. In contrast, the lack of fit (LOF) was statistically insignificant (*p* > 0.05). The coefficients of determination R^2^ and R^2^ adjusted have values above 0.9, which proves a high correlation of input and response data. 

However, not all factors of the obtained mathematical models were statistically significant, and the coefficient of the R^2^ predicted had a low value. Therefore, a reduction of statistically insignificant parameters was made, allowing for the simplification of the model while maintaining its statistical significance. The results of the analysis for the reduced models are presented in Table 3. 

The reduction of statistically insignificant components resulted in an improvement of the R^2^ predicted coefficient for extraction yield from 0.4985 to 0.8429, and for the content of fatty acids in the extract from 0.6123 to 0.8325. In addition, in the case of reduced models, a very low value of the “*p*” parameter was obtained, confirming that the developed models are statistically significant. The lack of fit is statistically insignificant in both cases. It can therefore be concluded that the developed mathematical models are suitable for describing the extraction yield and the content of fatty acids in the extract, within the established range of input parameters variability.

A statistical analysis of the regression coefficients showed that the significant expressions of the model for the extraction yield of blackcurrant seeds are the linear term of pressure (*X*_2_) and extraction time (*X*_3_), and the quadratic terms of pressure (*X*_2_^2^) and time (*X*_3_^2^). In contrast, significant model expressions for the fatty acid content of the extract are the linear term of pressure (*X*_2_), interactions between temperature and pressure (*X*_1_*X*_2_), interactions between pressure and extraction time (*X*_2_*X*_3_), and quadratic terms of pressure (*X*_2_^2^) and time (*X*_3_^2^).

**Figure 1:**
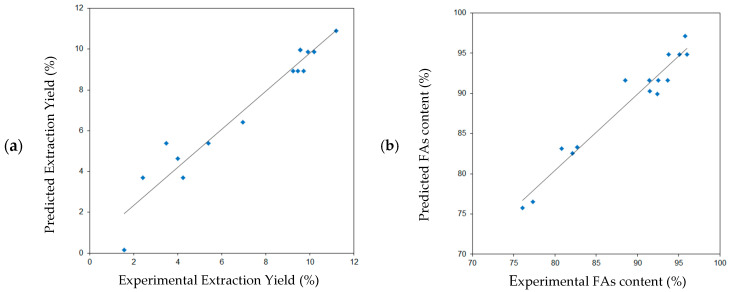
Comparison of the experimental results with the results predicted according to the developed mathematical models for: (**a**) the extraction yield (y = 0.9357x + 0.4571), (**b**) the content of fatty acids in the blackcurrant extract (y = 0.9506x + 4.3776).

Figure 1 shows, according to the developed model, the relationship between the predicted extraction yield of blackcurrant seeds (a) and the content of fatty acids in the extract (b) and experimental data.

The figures below show the response surface plots. 

**Figure 2:**
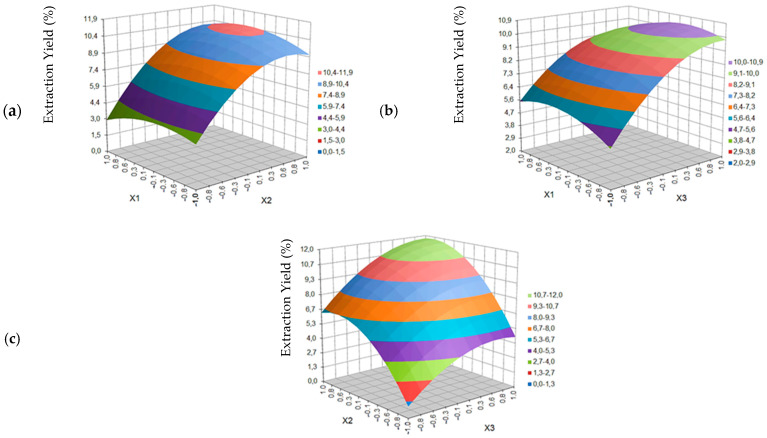
Response surface plot for blackcurrant seed extraction yield as a function of: (**a**) temperature (*X*_1_) and extraction pressure (*X*_2_), (**b**) temperature (*X*_1_) and extraction time (*X*_3_), (**c**) pressure (*X*_2_) and extraction time (*X*_3_).

Figure 2 shows the effect of temperature (*X*_1_), pressure (*X*_2_) and time (*X*_3_) on the yield of blackcurrant seed extraction (W_CP_) with supercritical carbon dioxide carried out in the ASFE MV-10 Waters system.

**Figure 3:**
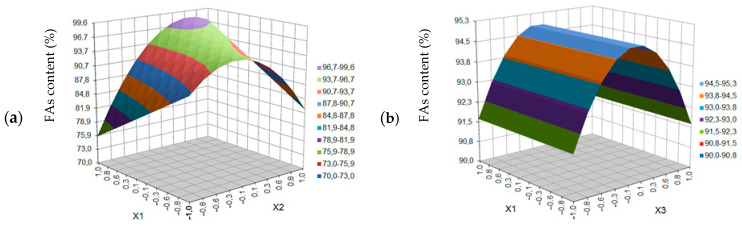

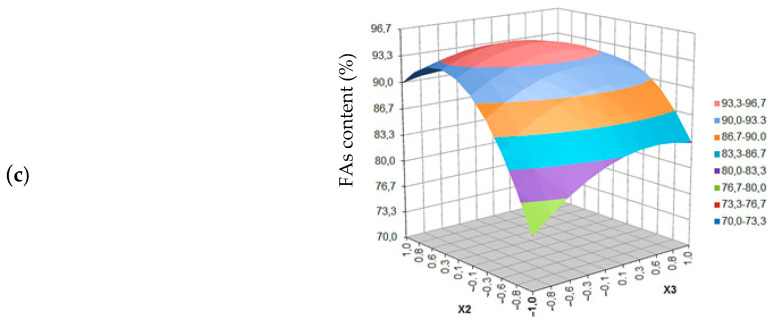
Response surface plot for fatty acid content in blackcurrant extract as a function of: (**a**) temperature (*X*_1_) and extraction pressure *(X*_2_), (**b**) temperature (*X*_1_) and extraction time (*X*_3_), (**c**) pressure (*X*_2_) and extraction time (*X*_3_).

The influence of the same extraction parameters on the content of fatty acids in the extract (C_FA_) is shown in Figure 3.

Below is the final form of the reduced mathematical model for the W_CP_ and C_FA_ as a function of coded (1) (3) and uncoded (2) (4) variables, respectively.

The yield of blackcurrant seed extraction:

W_CP_ = 8.865 + 3.133*X*_2_ + 2.235*X*_3_ − 2.053*X*_2_^2^ − 1.258*X*_3_^2^ (1)

W_CP_ = −13.96 + 0.126p + 0.269t − 0.000205p^2^ − 0.00315t^2^ (2)

Total content of fatty acids in blackcurrant seed extract:

C_FA_ = 94.836 + 3.403*X*_2_ + 7.270*X*_1_*X*_2_ − 8.398*X*_2_^2^ − 3.305*X*_2_*X*_3_ − 3.211*X*_3_^2^ (3)

C_FA_ = 41.91 + 0.405p + 0.00023Tp − 0.00082p^2^ − 0.00027pt + 0.00007t^2^ (4)

### 2.2. Black Cumin Seeds Extraction

Both the codes of the selected parameters, the equation describing the polynomial, and the statistical analysis of the results have the same wording as in the above description of blackcurrant seeds extraction. 

Table 4 presents the complete experimental design matrix and the results of the extraction of black cumin seeds with supercritical carbon dioxide obtained during the Box–Behnken design (BBD) implementation. The analyzed response variables were: W_BC_–extraction yield (%) and C_FA_–total fatty acid content (% *w/w*).

On the basis of the experimental data contained in Table 4, the regression coefficients of equations in the form of second-order polynomials for the extraction yield of black cumin seeds with supercritical carbon dioxide (W_BC_) and for the total content of fatty acids in the extracts (C_FA_) were determined, and a statistical analysis of these models was performed. Statistical analysis results for full quadratic models are presented in Table 5.

The *p*-value in the case of a mathematical model in the form of a second-order polynomial for the extraction yield of black cumin seeds and the content of fatty acids is 0.0042 and 0.0025, respectively. This means that the obtained models are statistically significant. The coefficient of determination R^2^ and R^2^ adjusted have a value of ≥0.90, which indicates a high correlation of input and response data. 

However, not all factors of the obtained mathematical models for the extraction yield of black cumin seeds and the content of fatty acids were statistically significant, as in the case of blackcurrant. Therefore, a reduction of statistically insignificant parameters was also made here, allowing for the simplification of the model, while maintaining its statistical significance. The results of the analysis for the reduced models are presented in Table 6. 

The reduction of statistically insignificant components resulted in an improvement of the R^2^ predicted coefficient for extraction yield from 0.4259 to 0.8271, and for the content of fatty acids from 0.6265 to 0.8686. In the case of the reduced model, a small *p*-value was obtained, confirming that the developed models are statistically significant. In addition, the lack of fit was statistically insignificant. Similar results for black cumin extraction yield (R^2^ adjusted 0.909; R^2^ predicted 0.877) were presented by Gawron et al. [24].

Statistical analysis of the regression coefficients showed that the significant expressions of the model for the extraction yield of black cumin seeds are the linear terms of pressure (*X*_2_) and extraction time (*X*_3_), in addition to the quadratic terms of pressure (*X*_2_^2^) and time (*X*_3_^2^). In contrast, the significant model expressions for the fatty acid content of the extract are the linear terms of pressure (*X*_2_) and extraction time (*X*_3_), temperature and time interactions (*X*_1_*X*_3_), pressure and time interactions (*X*_2_*X*_3_), and the quadratic term of pressure (*X*_2_^2^).

**Figure 4:**
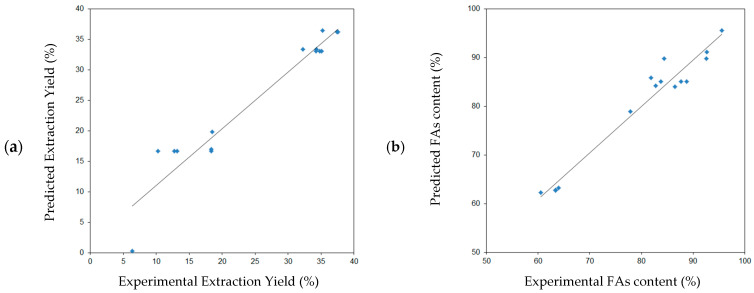
Comparison of the experimental results with the results predicted according to the developed mathematical models: (**a**) for extraction yield (y = 0.9300x + 1.7679), (**b**) for the content of fatty acids in the black cumin seed extract (y = 0.9541x + 3.6855).

According to the developed model, the relationship between the predicted extraction yield of black cumin seeds (a) and the content of fatty acids in the extract (b) and the experimental data are shown in Figure 4.

**Figure 5:**
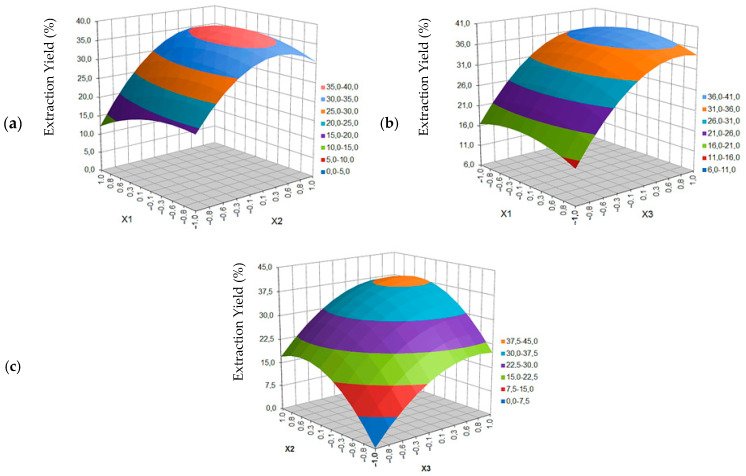
Response surface plot for black cumin seeds extraction yield as a function of: (**a**) temperature (X_1_) and extraction pressure (*X*_2_), (**b**) temperature (*X*_1_) and extraction time (*X*_3_), (**c**) pressure (*X*_2_) and extraction time (*X*_3_).

**Figure 6:**
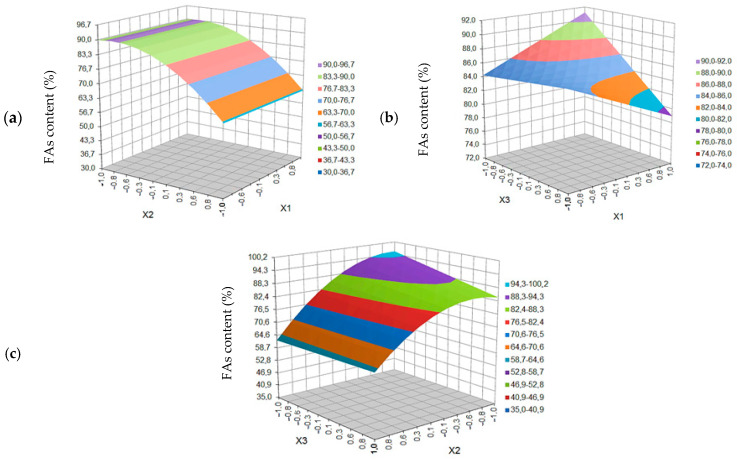
Response surface plot for fatty acid content in black cumin extract as a function of: (**a**) temperature (*X*_1_) and extraction pressure (*X*_2_), (**b**) temperature (*X*_1_) and extraction time (*X*_3_), (**c**) pressure (*X*_2_) and time extraction (*X*_3_).

Figure 5 shows the surface plot for black cumin seed extraction yield (W_BC_) as a function of variables *X*_1_*, X*_2_ and *X*_3_. The influence of temperature, pressure and extraction time on the content of fatty acids in the extract (C_FA_) is shown in Figure 6.

The final reduced mathematical models for response variables W_BC_ and C_FA_ in coded form (5) (7) and uncoded form (6) (8) are presented below.

Black cumin seeds extraction yield:

W_BC_ = 33.094 + 8.330*X*_2_ + 9.756*X*_3_ − 8.072*X*_2_^2^ − 6.650*X*_3_^2^ (5)

W_BC_ = −51.35 + 0.4546p + 1.319t − 0.0008p^2^ − 0.0166t^2^ (6)

Total content of fatty acids in black cumin seeds extract:

C_FA_ = 85.053 − 13.490*X*_2_ − 2.645*X*_3_ − 3.470*X*_1_*X*_3_ + 3.135*X*_2_*X*_3_ − 8.773*X*_2_^2^ (7)

C_FA_ = 81.98 + 0.2295p − 0.2378t − 0.0051Tt + 0.0016pt − 0.0009p^2^ (8)

## 3. Discussion

### 3.1. Blackcurrant Seeds Extraction

#### 3.1.1. Extraction Yield

As is shown in Figure 2a presenting the dependence of the process yield (W_CP_) as a function of temperature (T) and pressure (p), increasing the temperature value in one area of constant pressure (p) does not practically change the yield value. The increase in the yield is evident with the increase in pressure, which can be explained by the simultaneous increase of the solvent density. According to the literature [25,26,27], the higher the CO_2_ density, the higher the solubility of compounds and chemical substances contained in the test material. On the other hand, the lower the pressure and the higher the temperature, the CO_2_ will have a lower density, but also a higher diffusion coefficient and permeability. Too high a diffusion coefficient may then hinder or exclude fractionation, because compounds with different solubility (e.g., essential oils and glycerol esters of fatty acids) mix easily.

In the case of the dependence of extraction yield on temperature (T) and process time (t) (Figure 2b), increasing the value of T at constant time (similar at constant p), does not change the yield value. However, at low temperatures, (T) time is more important for process yield, while at high temperatures it is not so significant. Increasing the extraction time from 5 to 25 min, with constant values in the low temperature range (40–46 °C or <50 °C) increases the yield by more than 115% (from 3.8–4.7 to 8.2–9.1), while in the same period of time, i.e., 5–25 min at constant values in the high temperature range (54–60 °C or >50 °C), the yield increases by only 46% (from 5.6–6.4 to 8.2–9.1). In the range from 25 to 45 min in both temperature ranges, the yield increase is the lowest, but it is more than 21% (from 8.2–9.1 to 10.0–10.9).

From Figure 2c we can see the influence of pressure (p) and time (t) on the extraction yield of blackcurrant seeds. The lower the values of both parameters, evidently the lower the yield is. Increasing the pressure value (in a constant time range) will increase the yield faster than extending the time by running the process in a constant pressure. The highest yield values are expected in the time area above 25 min and at a pressure above 230 bar.

The optimal process parameters for the extraction yield, as determined on the basis of the developed model, are a pressure of 306 bar, a time of 43 min, and a temperature of 50 °C. The obtained W_CP_ value is 11.05%.

#### 3.1.2. Fatty Acids Content

As can be seen in Figure 3a, showing the dependence of the total content of fatty acids (C_FA_) as a function of temperature (T) and pressure (p), increasing the pressure value to approx. 230 bar, both at low and high temperatures, causes an overall increase in FAs content in the obtained oil extracts. The maximum is obtained in the range of pressure changes of 230 ± 25 bar, as well as temperature changes of 55 to 60 °C. Keeping the pressure value constant above 230 bar, but lowering the temperature from 55 to 40 °C, the FAs content decreases slightly, maintaining a constant level above 93.7% over a large area. By increasing the pressure to 230 bar, the fatty acid content changes over a wider percentage range (from 75.9 to 96.7–99.6) at higher temperature (60 °C) than at a lower one of 40 °C (from 81.9–84.8 to 93.7–96.7). An increase in pressure causes a faster increase in the total FAs content at lower temperatures.

In the case of the dependence of the FAs content on the temperature (T) and process time (t) (Figure 3b), increasing the temperature at a constant extraction time does not significantly change the total acid content. The temperature parameter turns out to be statistically insignificant. At 40 °C and 50 °C or 60 °C, the extraction time has the same effect on the FAs content: the longer the 5–25 min section, the higher the FAs content. However, after exceeding half of the experimentally planned time (from 25 to 45 min), a symmetrical decrease in the acids content is observed. Nevertheless, the yield of the process in the same section increases by more than 19% (from 9.1 to 10.9), which results from the possibility of extracting biological compounds other than FAs.

From Figure 3c, we can read the dependence of the FAs content on the mutual correlation between changes in pressure (p) and changes in the extraction time (t). The lower the values of both parameters, the lower the acids content. Increasing the pressure value (in a constant time range) will increase the FAs content faster than extending the time by running the process at a constant pressure. The highest values of FAs content (above 90.7%) were obtained in the pressure area above 200 bar, regardless of time.

The optimal process parameters for the content of fatty acids in the blackcurrant seed extract are a pressure of 301 bar, a time of 18 min, and a temperature of 60 °C. The C_FA_ value obtained then is 98.61% *w/w*.

### 3.2. Black Cumin Seeds Extraction

#### 3.2.1. Extraction Yield

As is shown in Figure 5a, presenting the dependence of the process yield (W_BC_) as a function of temperature (T) and pressure (p) and increasing the value (T) in one area of constant pressure does not significantly change the extraction yield, generating a slightly lower value at a higher temperature. However, with the increase in pressure, an increase in the extraction yield is observed, which can be explained, as in the case of blackcurrant, by the increase in the density of the solvent (CO_2_). The maximum yield is recorded for the temperature range of 45–55 °C and the pressure of 240–320 bar.

In the case of the dependence of extraction yield on temperature (T) and process time (t) (Figure 5b), increasing the value of (T) at constant time (similar at constant p) does not significantly change the yield value. On the other hand, extraction yield increases with time increase. This increase is faster in the range from 5 to 25 min regardless of the temperature. The maximum yield value is recorded for the temperature range of 43–57 °C and the time of the process from about 30 min.

In Figure 5c we can see the dependence of the W_BC_ value on the mutual correlations between changes in pressure (p) and changes in the extraction time (t). The lower the values of both parameters, the lower the yield evidently is. Both the pressure and the time changes, in the indicated ranges of the BBD experiments (130–330 bar and 5–45 min), generate a comparable change in yield value of 200% (from 0.0–7.5% to 15.0–22.5%). The yield values above 30%, for both 330 bar and 230 bar, can only be observed from 22 and 25 min, respectively.

The optimal process parameters for the extraction yield are the pressure of 282 bar, a time of 40 min and a temperature of 50 °C. The W_BC_ value obtained then is 38.82%. 

In general, the results of the implementation of the BBD plan show that the extraction yield of black cumin seeds ranges from 6.34 to 37.58%, depending on the parameters of the experiment. The average value for the eight higher values is 35.11%. The mean value is 13.96% for seven samples taken either at low pressure (130 bar) or at a short time (5 min). Similar extraction yield values (6.13–31.15%) were presented by Gawron et al. (2021) [1] in his publication. The lower values of 12% (at 250 bar, 60 °C) and 23.2% (at 350 bar, 60 °C, 120 min) were presented by Salea et al. (2013) and Solati et al. (2012), respectively [23,28].

#### 3.2.2. Fatty Acids Content

As can be seen from Figure 6a, showing the dependence of the total content of fatty acids (C_FA_) as a function of temperature (T) and pressure (p) for black cumin extract samples, increasing the pressure value to 330 bar, both at low and high temperature, causes a general symmetrical decrease in the content of FAs in the obtained oil extracts to approx. 56.7–63.3% *w/w*. The maximum C_FA_ (90.0–96.7% *w/w*) is obtained in a fairly narrow range of pressure changes, from 163 ± 11 bar, but at any temperature in the range of 40–60 °C. By keeping the pressure value at any level between 240 and 130 bar, a high content of FAs (above 83.3%) is maintained, regardless of the temperature in the range of 40–60 °C.

The effect of temperature (T) and time (t) on the content of fatty acids in the black cumin seeds extracts is shown in Figure 6b. A statistical analysis showed that the interactions between these parameters are important for the content of FAs in this extracts. An increase in temperature causes an increase in the fatty acid content in a short extraction time. However, with a longer extraction time, a decrease in the FAs content in the extracts with increasing temperature is observed. For experiments conducted at higher temperature, the content of fatty acids decreased with the increase of extraction time.

The dependence of the FAs content on the mutual correlation between changes in pressure (p) and changes in extraction time (t) is shown at Figure 6c. The higher the pressure values and the longer the extraction time, the more pronounced the decrease in the acids content. The maximum expected FAs content covers the area of lowest pressure (130–174 bar) and short extraction time (5–10 min). Subsequently, increasing the time and pressure above these values, the fatty acid content decreases, and in the same pressure area, increasing the time does not cause any changes in C_FA_.

The optimal process parameters for the content of fatty acids in the black cumin seed extract are the pressure of 135 bar, time of 5 min and temperature of 60 °C. The C_FA_ value obtained is 99.04%.

### 3.3. Other Dependencies Affecting the Obtained Results

The obtained results can be used to transfer the experiment to a larger scale (e.g., quarter–technical). However, when comparing the obtained values, one should remember the homogeneity and amount (mass) of the extraction sample, as well as all processes and interactions between the supercritical phase and the liquid and gas phases, which are difficult to define.

Although the plant material may come from one purchase lot, a complete transfer in the results will not be accurately reflected from one scale to another. Even at a certain short distance of the operating point (40 °C) from the critical point (for CO_2_ it is T_c_ = 31.1 °C and p_c_ = 73.8 bar), scientists notice various properties of the supercritical fluid, which may affect the kinetics and dynamics of the extraction process and thus change the solubility of the substances contained in the sample matrix [26,29,30].

As is known, plant oil extracts usually consist of many components with different structures and different physicochemical properties. These components, being both pure substances and mixtures, show different solubility depending on the solvent and process parameters (pressure, temperature, time, mas transfer, density). Due to this different solubility of individual compounds contained in the extracts, it is possible to partially separate them. As a result of this process, plant oil fractions enriched or depleted in certain bio-components can already be obtained at the production stage [29]. 

By changing the parameters of the scCO_2_ extraction process, it is possible to modify the composition of the obtained oil, striving to obtain the maximum amount of product (emphasizing extraction yield–Table 1 and Table 4) or obtaining a product with a high content of the selected ingredient (specific saturated/unsaturated FA–Table 7 and Table 8).

The observed increase in the total FAs content with increasing pressure, at constant temperature and constant extraction time, can be explained by the fact that higher pressure increases the density of the solvent (CO_2_), and this results in higher solvation capacity in supercritical carbon dioxide. As a consequence, the greater dissolution of oil components occurs at higher pressure [27], as in the case of seeds extraction of blackcurrant (Figure 3a,c, pressure from 130 to 230 bar) and black cumin (on a shorter section of pressure changes, 130–174 bar).

However, the research showed that such a process may run up to certain pressure values and then the reverse relation may occur. With the increase in pressure the content of extracted components may decrease (in this case, the sum of FAs). This is because, in addition to the general process conditions, the solute’s solubility in the supercritical solvent is also determined by its vapor pressure, molecular weight, and polarity. The higher vapor pressure of the solute facilitates its removal from the matrix [31]. In turn, as the molecular weight of a solute increases, its solubility decreases.

As reported by Güçlü-Üstündag O. and Temelli, F. (2000) [25], in a homologous series of compounds, the solubility decreases with the increasing number of carbon atoms or molecular weight. For example, during scCO_2_ extraction at 35 °C and increasing pressure, from two tested FAs, the oleic acid (282 g/mol) was more soluble than stearic acid (284 g/mol). This increase in solubility was due to the presence of a double bond in the structure of oleic acid, which significantly lowers its melting point. However, further studies also showed that the effect of the presence of a double bond on solubility is mainly due to the difference in the physical state of the solute (at 35 °C, oleic acid is a liquid, and stearic acid is a solid). When both solutes were in the liquid state, their solubilities were similar.

An increase in solubility with a decrease in molecular weight, however, cannot be observed in a series of unsaturated FAs or their esters, where the introduction of a double bond only slightly changes the solubility. According to Gustinelli G. et al. (2018), [32] α-linolenic acid (18:3, ω3) is found mainly in the triacylglycerol fraction, and therefore its concentration depends on the solubility of triacylglycerols, which in turn depends on their molecular weight. About 26 different types of triacylglycerols have been described for blackcurrant seed oil, which does not make it easier for analysts to interpret the obtained results (Johansson et al. 1997) [33].

Similarly, the presence of polar groups in the solute structure reduces its solubility in non-polar supercritical carbon dioxide. At low solvent density, the molar mass effect of the solute dominates, giving way to the polarity effect as the density of the solvent increases [25]. Free fatty acids (FFA), mono- and diglycerides are more soluble in scCO_2_ than triglycerides [34]. The solubility of mono- and diglycerides is between FFA and triglycerides [35]. Yu et al. (1994) [27] suggested that the molecular weight of fatty acids (carbon chain length) is a more important factor affecting solubility than the degree of unsaturation. In some cases, the higher pressure used in the scCO_2_ extraction resulted in oils with a higher content of ω-3 and ω-6 acids than the lower pressure at a constant temperature (Table 7—blackcurrant, experiments no. 1 and 15, 7 and 8, 5 and 14, and 8 and 11). Cheung (1999) [36] reported similar conclusions in his research. The opposite situation is observed in the presented studies with the extraction of black cumin seeds, as a typical oily plant, where the PUFA content is higher for samples obtained at lower pressure (Table 8).

In mixtures of similar compounds, the intermolecular interactions are similar, and the vapor pressure of the solute depends on the molecular weight of the compounds [26,29]. However, in complex mixtures, such as oils from berry seeds or oilseeds, the complex structure and complex intermolecular interactions cause significant differences compared to the solubility of pure lipids (fatty acids and mono-, di- and triacylglycerols) [25]. Therefore, it is more difficult to predict the individual vapor pressures and their effect on the extraction yield and product composition from different seeds extracted, even under the same conditions [32].

## 4. Materials and Methods

### 4.1. Plant Material

As a model raw material for research, the seeds of two different plants were selected. One was the blackcurrant (*Ribes nigrum* L.)–a berry, perennial plant from the gooseberry family, while the other was the black cumin (*Nigella sativa* L.), an annual oil plant from the buttercup family. Both species have been cultivated for utility purposes since the 15th–16th centuries, and their medicinal properties, as plants in the wild, were used even earlier.

**Figure 7:**
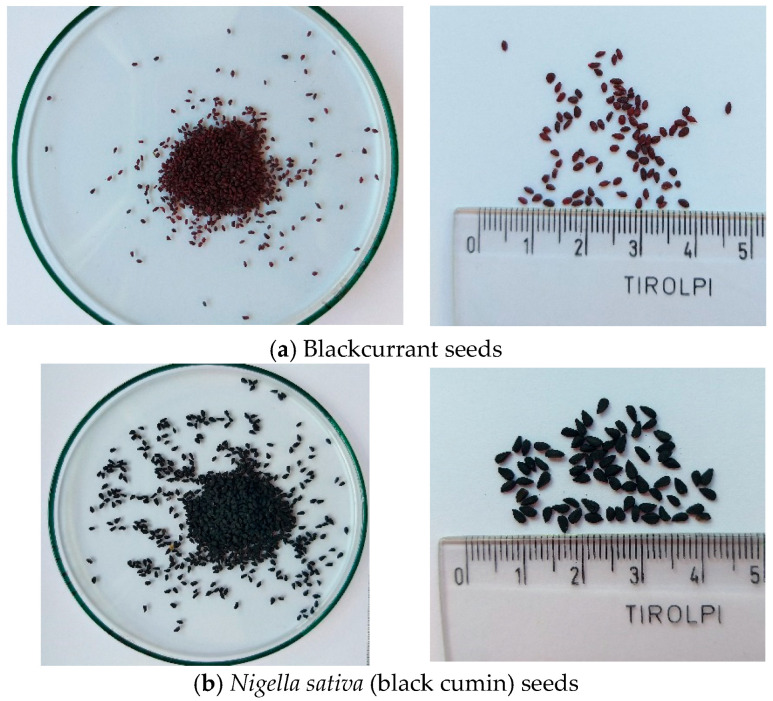
Blackcurrant and black cumin seeds used in the study of the supercritical extraction process (Source: author’s photo). (**a**) Blackcurrant seeds, ellipsoidal in shape, 1–2 mm in size, brown-purple in colour. (**b**) *Nigella sativa* (black cumin) seeds, pyramidal shape with a clear sharp tip at one end, size 2–3 mm, intense black color.

All material was purchased from Dary Natury, Koryciny (black cumin seeds) and Zielony Klub, Kielce (blackcurrant seeds). The seeds were ground before extraction in an IKA laboratory mill. The appearance of the seeds is shown in Figure 7.

### 4.2. Supercritical Extraction on a Laboratory Scale 

**Figure 8:**
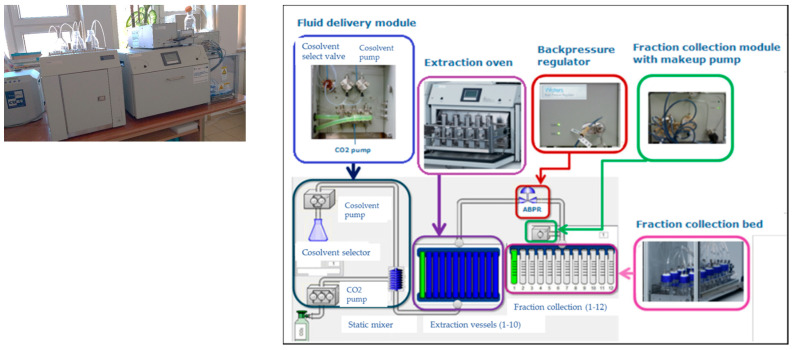
The external appearance and the component diagram of a Waters ASFE MV-10 extractor (Source: author’s photo; diagram MV-10 ASFE System Guide for ChromScope IE v1.20).

The extraction process was carried out in the Chromatographic Laboratory of the Research Laboratory of Plant Raw Materials and Products of the Łukasiewicz Research Network–New Chemical Syntheses Institute. A Waters SFE MV-10 laboratory supercritical extractor (Waters Corporation, Milford, MA, USA) presented in Figure 8 was used for this purpose.

The process begins with filling the prepared sample (ground or crushed) into the extraction vessel. The vessel is then placed in the extraction oven and the selected parameters are set. After reaching the set values of T, p and CO_2_ flow, the valves are automatically opened and the extraction is started. After the process is completed, the system automatically goes to the balancing stage, during which the ABPR valve (back pressure regulator) reduces the system pressure to atmospheric pressure. The obtained extract flows directly into the fraction collector.

The scale of extraction is smaller, which allows the use of a small amount of sample (several grams) and less media consumption. A small amount of the obtained extract, usually a few milliliters, can sometimes be as a disadvantage of this technique, but this amount depends on the type of matrix and extraction parameters.

### 4.3. Experimental Design of Oil Extraction 

The extraction process of the selected seeds was carried out using the Box–Behnken design (BBD) for three independent variables: temperature of 40, 50 and 60 °C; pressure values of 130, 230 and 330 bar, and extraction time of 5, 25 and 45 min, respectively. The weight of a single charge into the 5 mL extraction vessel was 7.0 g and 3.6 g for black currant and black cumin seeds, respectively. The carbon dioxide flow for both matrices was set at 7 mL/min to allow the obtained results comparison.

Carrying out the process under the coded parameter given in Table 9, a series of oil extracts from black currant and black cumin seeds were obtained. As a result, the percentage extraction yield and the fatty acid content were determined after the end of the process. 

The influence of the extraction parameters of both seeds on the response variables was approximated by a second-order polynomial regression model described by the following equation:

$$y=\beta_{0}+\beta_{1}X_{1}+\beta_{2}X_{2}+\beta_{3}X_{3}+\beta_{12}X_{1}X_{2}+\beta_{13}X_{1}X_{3}+\beta_{23}X_{2}X_{3}+\beta_{11}X_{1}^{2}+\beta_{22}X_{2}^{2}+\beta_{33}X_{3}^{2}$$

where: y–response variable, *X*_1_, *X*_2_, *X*_3_–independent variables, respectively: temperature (T), pressure (p) and time (t), *β*–coefficients in the equation defining: equation constant (*β*_0_), main effects of parameters (*β*_1_, *β*_2_, *β*_3_), their interaction (*β*_12_, *β*_23_, *β*_13_) and quadratic terms (*β*_11_, *β*_22_, *β*_33_).

The analysis of variance (ANOVA) was used for the statistical evaluation of the obtained results. The F test was performed to assess the significance of the estimated coefficients in the model. The test of statistical significance was based on error criteria with a confidence level of 95%. A regression analysis was performed using Design Expert 11.0.6.0 (Stat-Ease Inc., Minneapolis, MN, USA).

### 4.4. Gas Chromatography of Fatty Acids

The FA analysis method consists in the indirect determination of the content of individual fatty acids (FA) in the form of methyl esters (FAMEs) obtained by esterification with methanolic solution of trimethylsulfonic hydroxide (TMSH). Samples of both oil extracts were prepared according to ISO 12966, part 1–4. A 10 (±2) mg sample was dissolved in 500 µL of tert-butyl methyl ether and mixed for 5 min. Next, 250 µL of TMSH solution (0.25 M in methanol) was added, and the sample was shaken for 5 min to obtain methyl derivatives. The components were analyzed on GC/MSD. 

The quantitation analysis was based on analytical standards (from Sigma-Aldrich Co., St. Louis, MO, USA) and external calibration curves for each compound separately. The obtained FAMEs value were converted to FAs by molecular weight and dilution factor. 

The total yield of the extraction process was determined by gravimetric measurement of the mass of the obtained extract and expressed as a percentage of the initial mass of seeds constituting the extraction vessel charge.

A gas chromatography analysis was performed on Agilent equipment (GC 6890N, Agilent Technologies, Inc. Santa Clara, CA, USA) with a single quadrupole mass spectrometer detector (MSD 5975), split/splitless injector and an Agilent J&W GC capillary column type HP-88 for FAMEs separation. Both GC and MSD systems were controlled by MSD ChemStation, version E.02.02.1431 (Agilent Technologies, Inc. Santa Clara, CA, USA). 

**Figure 9:**
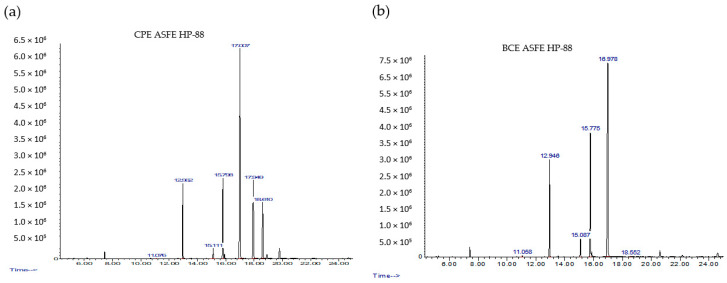
Chromatograms of FAs in blackcurrant (**a**) and black cumin (**b**) extracts.

The examples of fatty acid chromatograms obtained during chromatography analysis are presented in Figure 9 and Table 10.

## 5. Conclusions

The use of carbon dioxide in the supercritical state for the extraction of blackcurrant and black cumin seeds allowed for high process yield and high-quality, rich in polyunsaturated fatty acids oils which can be used as a substrate or final product in several industries.

All listed parameters, temperature, pressure and extraction time have a similar effect on the yield of blackcurrant and black cumin seeds extraction obtained in a laboratory supercritical extractor (ASFE MV-10 Waters). In both examples, the significant expressions of the model are the linear terms of pressure (*X*_2_) and extraction time *(X*_3_), and the quadratic terms of pressure (X_2_^2^) and time (*X*_3_^2^). An increase in pressure causes an increase in extraction yield (W), and an increase in temperature, both at constant pressure and time, does not significantly change the yield value. For both materials, the optimal yield values were obtained under almost similar extraction parameters: 306 bar/ 43 min/ 50 °C (blackcurrant) and 282 bar/ 40 min/ 50 °C (black cumin).

The influence of the above parameters (T, p, t) on the content of FAs in the extracts has a slightly different trend. In both examples, the significant expressions of the model are the linear term of pressure (*X*_2_), the quadratic term of pressure (*X*_2_^2^), and the pressure–time interactions (*X*_2_*X*_3_). However, in the case of black cumin, the interactions of temperature and time (*X*_1_*X*_3_) were also significant, and in the case of blackcurrant, the quadratic term of time (*X*_3_^2^) was also significant, which is reflected in the obtained response surface plots. The content of FAs in black cumin extracts decreases with increasing pressure and increasing extraction time. When the process temperature increases, the FAs content in black cumin extracts increases, but only when the extraction time is relatively short (up to about 30 min) and decreases when the time is longer. 

## Figures and Tables

**Table 1:** Extraction parameters in individual experiments and the results of extraction of black-currant seeds with supercritical carbon dioxide on the ASFE MV-10 Waters extractor.

Run Order	Temperature, °C	*X*_1_	Pressure, bar	*X*_2_	Time, min	*X*_3_	W_CP_ (%)	C_FA_ (% *w/w*)
1	50	0	130	−1	5	−1	1.57	77.34
2	40	−1	230	0	45	1	9.90	92.52
3	40	−1	330	1	25	0	9.56	82.11
4	50	0	330	1	45	1	11.19	82.67
5	40	−1	230	0	5	−1	3.48	93.67
6	50	0	230	0	25	0	9.71	95.99
7	60	1	330	1	25	0	9.56	95.76
8	60	1	130	−1	25	0	2.40	76.06
9	50	0	230	0	25	0	9.24	93.79
10	50	0	230	0	25	0	9.13	95.06
11	60	1	230	0	45	1	10.18	91.44
12	60	1	230	0	5	−1	5.38	88.54
13	50	0	130	−1	45	1	3.99	80.83
14	40	−1	130	−1	25	0	4.24	91.49
15	50	0	330	1	5	−1	6.95	92.40

W_CP_—yield for the extraction of blackcurrant seeds, %. C_FA_—total content of fatty acids in samples of oil extracts from blackcurrant seeds, % *w/w*.

**Table 2:** Regression coefficients of the full square models with their statistical significance and model parameters for blackcurrant seed extraction yield (W_CP_) and fatty acid content (C_FA_).

Source	W_CP_ (%)		C_FA_ (% *w/w*)	
	Regression Coefficient, *β*	*p*-Value	Regression Coefficient, *β*	*p*-Value
Model		0.0032		0.0021
LOF		0.0586		0.1933
*β*_0_	9.360		94.95	
*X*_1_	0.042	0.9072	−0.999	0.1997
*X*_2_	3.133	0.0003	3.403	0.0040
*X*_3_	2.235	0.0013	−0.561	0.4443
*X*_1_ *X*_2_	0.460	0.3913	7.270	0.0006
*X*_1_ *X*_3_	−0.405	0.4465	1.013	0.3381
*X*_2_ *X*_3_	0.455	0.3961	−3.305	0.0181
*X*_1_^2^	−0.805	0.1756	−0.180	0.8639
*X*_2_^2^	−2.115	0.0090	−8.412	0.0004
*X*_3_^2^	−1.320	0.0491	−3.225	0.0229
R^2^		0.9676		0.9726
R^2^ adjusted		0.9092		0.9234
R^2^ predicted		0.4985		0.6123

*p* < 0.0001 very highly significant, *p* < 0.01 very significant, *p* < 0.05 significant, *p* > 0.10 insignificant, LOF (Lack of Fit)–*p*-value of the model non-fit test.

**Table 3:** Regression coefficients of the reduced mathematical models with their statistical significance and model parameters for blackcurrant seed extraction yield (W_CP_) and fatty acid content (C_FA_).

Source	W_CP_ (%)		C_FA_ (% *w/w*)	
	Regression Coefficient, *β*	*p*−Value	Regression Coefficient, *β*	*p*−Value
Model		<0.0001		<0.0001
LOF		0.1493		0.4187
*β*_0_	8.865		94.836	
*X*_1_				
*X*_2_	3.133	<0.0001	3.403	0.0007
*X*_3_	2.235	0.0001		
*X*_1_ *X*_2_			7.270	<0.0001
*X*_1_ *X*_3_				
*X*_2_ *X*_3_			−3.305	0.0073
*X*_1_^2^				
*X*_2_^2^	−2.053	0.0023	−8.398	<0.0001
*X*_3_^2^	−1.258	0.0325	−3.211	0.0103
R^2^		0.9356		0.9506
R^2^ adjusted		0.9098		0.9232
R^2^ predicted		0.8453		0.8325

*p* < 0.0001 very highly significant, *p* < 0.01 very significant, *p* < 0.05 significant, *p* > 0.10 insignificant, LOF (Lack of Fit)—*p*-value of the model non-fit test.

**Table 4:** Extraction parameters in individual experiments and the results of extraction of black cumin seeds with supercritical carbon dioxide on the ASFE MV-10 Waters extractor.

Run Order	Temp. °C	*X*_1_	Pressure, bar	*X*_2_	Time, min	*X*_3_	W_BC_ (%)	C_FA_ (% *w/w*)
1	50	0	130	−1	5	−1	6.34	95.55
2	40	−1	230	0	45	1	37.42	81.89
3	40	−1	330	1	25	0	32.28	63.41
4	50	0	330	1	45	1	35.19	63.97
5	40	−1	230	0	5	−1	13.18	82.75
6	50	0	230	0	25	0	34.83	87.62
7	60	1	330	1	25	0	34.28	63.32
8	60	1	130	−1	25	0	10.28	84.43
9	50	0	230	0	25	0	35.07	88.79
10	50	0	230	0	25	0	34.26	83.80
11	60	1	230	0	45	1	37.58	77.89
12	60	1	230	0	5	−1	12.72	92.63
13	50	0	130	−1	45	1	18.47	86.50
14	40	−1	130	−1	25	0	18.38	92.58
15	50	0	330	1	5	−1	18.37	60.48

W_BC_—yield for the extraction of black cumin seeds, %. C_FA_—total content of fatty acids in samples of oil extracts from black cumin seeds, % *w/w*.

**Table 5:** Regression coefficients of the full quadratic models with their statistical significance and model parameters for black cumin seed extraction yield (W_BC_) and fatty acid content (C_FA_).

Source	W_BC_ (%)		C_FA_ (% *w/w*)	
	Regression Coefficient, *β*	*p*-Value	Regression Coefficient, *β*	*p*-Value
Model		0.0042		0.0025
LOF		0.0079		0.3315
*β*_0_	34.720		86.737	
*X*_1_	−0.800	0.5601	−0.295	0.8160
*X*_2_	8.330	0.0013	−13.490	0.0001
*X*_3_	9.756	0.0006	−2.645	0.0791
*X*_1_ *X*_2_	2.525	0.2224	2.015	0.2893
*X*_1_ *X*_3_	0.155	0.9352	−3.470	0.0968
*X*_2_ *X*_3_	1.173	0.5463	3.135	0.1246
*X*_1_^2^	−2.642	0.2204	−1.818	0.3514
*X*_2_^2^	−8.275	0.0071	−8.983	0.0038
*X*_3_^2^	−6.853	0.0150	−1.128	0.5519
R^2^		0.9640		0.9707
R^2^ adjusted		0.8991		0.9180
R^2^ predicted		0.4259		0.6265

*p* < 0.0001 very highly significant, *p* < 0.01 very significant, *p* < 0.05 significant, *p* > 0.10 insignificant, LOF (Lack of Fit)—*p*-value of the model non-fit test.

**Table 6:** Regression coefficients of the reduced mathematical models with their statistical significance and model parameters for black cumin seed extraction yield (W_BC_) and fatty acid content (C_FA_).

Source	W_BC_ (%)		C_FA_ (% *w/w*)	
	Regression Coefficient, *β*	*p*-Value	Regression Coefficient, *β*	*p*-Value
Model		<0.0001		<0.0001
LOF		0.0668		0.6281
*β*_0_	33.094		85.053	
*X*_1_				
*X*_2_	8.330	0.0001	−13.490	<0.0001
*X*_3_	9.756	<0.0001	−2.645	0.0428
*X*_1_ *X*_2_				
*X*_1_ *X*_3_			−3.470	0.0565
*X*_2_ *X*_3_			3.135	0.0796
*X*_1_^2^				
*X*_2_^2^	−8.072	0.0014	−8.773	0.0005
*X*_3_^2^	−6.650	0.0050		
R^2^		0.9300		0.9541
R^2^ adjusted		0.9020		0.9287
R^2^ predicted		0.8271		0.8686

*p* < 0.0001 very highly significant, *p* < 0.01 very significant, *p* < 0.05 significant, *p* > 0.10 insignificant, LOF (Lack of Fit)—*p*-value of the model non-fit test.

**Table 7:** The content of individual fatty acids in blackcurrant seed extracts (% *w/w*) vs BBD experiment parameters.

No.	T/p/t °C/bar/min	Palmitic Acid C16:0	Stearic Acid C18:0	Oleic Acid C18:1 ω9	Linoleic Acid C18:2 ω6	Gamma Linolenic Acid C18:3 ω6	Alfa Linolenic Acid C18:3 ω3	Sum (% *w/w*)
1	50/130/5	7.48	0.82	9.38	38.39	11.90	9.37	77.34
2	40/230/45	7.04	1.17	11.55	45.95	14.92	11.88	92.51
3	40/330/25	5.78	0.97	10.42	41.22	13.24	10.49	82.11
4	50/330/45	5.79	1.03	10.46	41.53	13.32	10.53	82.67
5	40/230/5	7.01	1.17	12.06	46.36	15.06	12.02	93.67
6	50/230/25	7.34	1.17	11.97	47.30	15.90	12.32	95.99
7	60/330/25	6.76	1.26	12.19	47.55	15.61	12.39	95.76
8	60/130/25	5.43	0.98	9.78	38.57	11.84	9.47	76.06
9	50/230/25	7.78	1.27	11.69	46.13	15.25	11.68	93.79
10	50/230/25	7.11	1.19	11.76	46.34	15.81	12.36	95.06
11	60/230/45	6.51	1.14	11.66	45.51	14.93	11.69	91.44
12	60/230/5	6.66	1.05	11.13	44.44	14.28	10.97	88.54
13	50/130/45	6.42	1.04	10.06	40.43	12.89	9.98	80.83
14	40/130/25	7.84	1.18	11.34	44.99	14.94	11.19	91.49
15	50/330/5	7.39	1.13	11.46	45.47	15.34	11.59	92.40

T—temperature, °C; p—pressure, bar; t—extraction time, min.

**Table 8:** The content of individual fatty acids in black cumin extracts (% *w/w*) vs. BBD experiment parameters.

No.	T/p/t °C/bar/min	Palmitic Acid C16:0	Stearic Acid C18:0	Oleic Acid C18:1 ω9	Linoleic Acid C18:2 ω6	Alfa Linolenic Acid C18:3 ω3	Sum (% *w/w*)
1	50/130/5	13.77	2.88	22.63	56.13	0.14	95.55
2	40/230/45	10.75	2.24	19.55	49.02	0.33	81.89
3	40/330/25	7.83	1.69	15.60	38.19	0.11	63.41
4	50/330/45	7.94	1.84	15.81	38.28	0.11	63.97
5	40/230/5	10.93	2.28	19.69	49.71	0.14	82.75
6	50/230/25	11.48	2.39	21.01	52.59	0.14	87.62
7	60/330/25	7.92	1.78	15.46	38.02	0.14	63.32
8	60/130/25	10.42	2.23	20.77	50.86	0.15	84.43
9	50/230/25	12.22	2.53	21.14	52.75	0.15	88.79
10	50/230/25	10.87	2.43	20.36	50.00	0.14	83.80
11	60/230/45	9.83	2.23	19.03	46.65	0.15	77.89
12	60/230/5	13.55	2.89	22.03	54.02	0.14	92.63
13	50/130/45	10.46	2.28	21.15	52.48	0.14	86.50
14	40/130/25	13.07	2.79	21.98	54.60	0.14	92.58
15	50/330/5	7.80	1.56	14.36	36.62	0.14	60.48

T—temperature, °C; p—pressure, bar; t—extraction time, min.

**Table 9:** Coded and uncoded values of the independent variables applied in the experimental design.

Coded Parameters		−1	0	+1
*X*_1_	T–temperature, °C	40	50	60
*X*_2_	p–pressure, bar	130	230	330
*X*_3_	t–time, min	5	25	45

**Table 10:** Fatty acids determined during chromatography analysis.

FA Name	RT, min CPE	RT, min BCE
Myristic acid	11.07	11.06
Palmitic acid	12.98	12.95
Stearic acid	15.11	15.09
Oleic acid	15.79	15.78
Linoleic acid	17.00	16.98
Gamma–Linolenic acid	17.95	-
Alfa –Linolenic acid	18.61	18.55

## Data Availability

The data presented in this study are available on request from the corresponding author.

## Sample Availability

Samples of the oils are available from the authors.
